# How to Tackle ADHD Medication Shortage and Changes in Shared Care Protocol (SCP) in Child and Adolescent Mental Health Services (CAMHS)

**DOI:** 10.1192/j.eurpsy.2025.1180

**Published:** 2025-08-26

**Authors:** S. M. Toparlak, E. Fergusson, M. Costa, C. Kowalski, R. Chapman, F. Whitaker, R. Hogan

**Affiliations:** 1 Oxford Health NHS Foundation Trust, Oxford, United Kingdom

## Abstract

**Introduction:**

Our team faced two challenges in prescribing for patients with ADHD. On 27 September 2023, a shortage of methylphenidate prolonged-release capsules and tablets, lisdexamfetamine capsules, and guanfacine prolonged-release tablets was announced in the UK.

SCP is an agreement between secondary care, patient and GPs, which asks GPs to continue prescribing ADHD medication when young person is stable on a dose, this also applies to when the doses are changed. However, many GP practices in Oxfordshire declared a position statement that they would decline prescribing ADHD medication to adult or child patients on a “shared care” basis due to funding issues to support specialist reviews. These challenges led us to adapt our practices and create a protocol.

**Objectives:**

To make all staff members aware of recent updates on ADHD medication stock availability at local depots to ensure continuity of care and minimise distress.

To help reduce the number of declined shared care requests by GPs in 6–17-year-olds.

To identify the patients of GP practices that do not prescribe ADHD medication to adults so that a planned discontinuation of treatment can take place prior to discharge from CAMHS services.

**Methods:**

Young people’s and their carers’ feedback regarding the medication shortage were carefully recorded.

Concerns were raised and discussed in multi-disciplinary meetings via MS Teams and in person.

Team members shared information with each other with a view to creating a protocol to minimise the risk to patients’ safety and distress in families.

**Results:**

Regular updates on ADHD medication availability, along with equivalent conversions of long-acting medications were sent to all staff members via email by the Trust.

Our service developed a “protocol for CAMHS ADHD when the children and young people’s (CYP) shared care protocol is declined and how to handle discharges at age 18.”

We agreed to make young people and carers aware of the current situation for prescribing ADHD medication in adults within Oxfordshire to explore the need for continuing medication in reviews.

ADHD medications were planned to be discontinued prior to discharge, if patients are 17, on ADHD medication, whose GP practice declines SCP. and do not meet threshold for community adult mental health team referral.

We agreed to consider prescribing for a brief time beyond age 18 on a case-by-case basis if patients are preparing for, or are currently taking, A-level (or other) exams or whereby discontinuing medication poses significant risk.

**Conclusions:**

Patients and staff members were greatly affected by the ADHD supply issues as well as current changes in SCP. However, team members took necessary steps to address the current issues proactively.

We plan to develop guidelines about stopping stimulants under supervision prior to discharge from CAMH services.

**Disclosure of Interest:**

None Declared

